# Effects of the Structure and Temperature on the Nature of Excitons in the Mo_0.6_W_0.4_S_2_ Alloy

**DOI:** 10.1021/acs.jpcc.1c09806

**Published:** 2022-01-25

**Authors:** Deepika Poonia, Nisha Singh, Jeff J. P. M. Schulpen, Marco van der Laan, Sourav Maiti, Michele Failla, Sachin Kinge, Ageeth A. Bol, Peter Schall, Laurens D. A. Siebbeles

**Affiliations:** † Optoelectronic Materials Section, Department of Chemical Engineering, Delft University of Technology, 2629 HZ Delft, The Netherlands; ‡ Department of Applied Physics, Eindhoven University of Technology, P.O. Box 513, 5600 MB Eindhoven, The Netherlands; § Institute of Physics, University of Amsterdam, 1098 XH Amsterdam, The Netherlands; ∥ Materials Research & Development, Toyota Motor Europe, B1930 Zaventem, Belgium

## Abstract

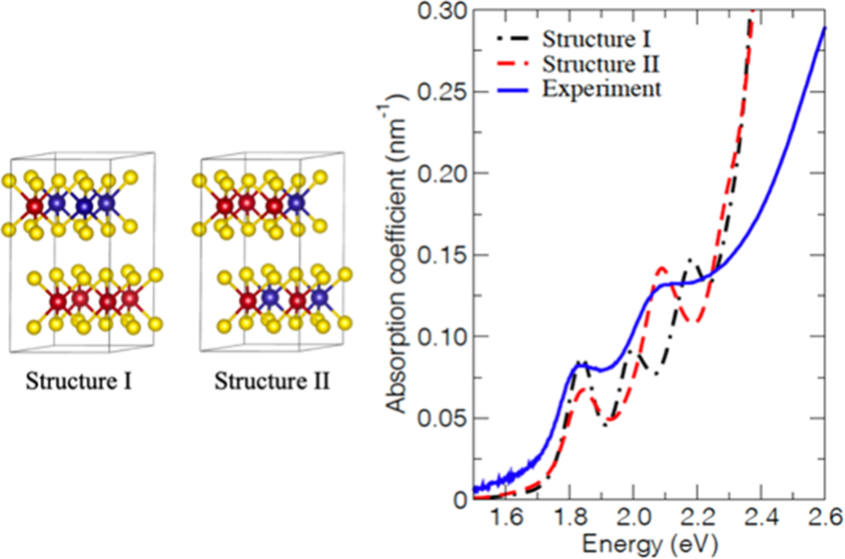

We studied the nature of excitons in the transition metal dichalcogenide alloy Mo_0.6_W_0.4_S_2_ compared to pure MoS_2_ and WS_2_ grown by atomic layer deposition (ALD). For this, optical absorption/transmission spectroscopy and time-dependent density functional theory (TDDFT) were used. The effects of temperature on A and B exciton peak energies and line widths in optical transmission spectra were compared between the alloy and pure MoS_2_ and WS_2_. On increasing the temperature from 25 to 293 K, the energy of the A and B exciton peaks decreases, while their line width increases due to exciton–phonon interactions. The exciton–phonon interactions in the alloy are closer to those for MoS_2_ than those for WS_2_. This suggests that exciton wave functions in the alloy have a larger amplitude on Mo atoms than that on W atoms. The experimental absorption spectra could be reproduced by TDDFT calculations. Interestingly, for the alloy, the Mo and W atoms had to be distributed over all layers. Conversely, we could not reproduce the experimental alloy spectrum by calculations on a structure with alternating layers, in which every other layer contains only Mo atoms and the layers in between also contain W atoms. For the latter atomic arrangement, the TDDFT calculations yielded an additional optical absorption peak that could be due to excitons with some charge transfer character. From these results, we conclude that ALD yields an alloy in which Mo and W atoms are distributed uniformly among all layers.

## 1 Introduction

Layered van der Waals materials, in particular transition metal dichalcogenides (TMDCs), have gained considerable interest due to prospects for applications in, e.g., photodetectors,^1,2^ sensors,^3,4^ and solar cells.^5−7^ These materials consist of layers in which transition metal atoms are covalently bound to chalcogen (S, Se, Te) atoms. The layers are stacked on top of each other and held together by van der Waals forces.^8,9^ TMDCs with chemical composition MX_2_ (M = Mo, W, etc., and X = S, Se) have been studied extensively owing to their direct band gap in monolayers,^10^ valley selective optical coupling,^11^ and large exciton binding energies.^12^ Alloying has been used to vary the relative content (*y*) of the transition metal or chalcogen atoms and obtain layers of M*_y_*M_1–*y*_^′^X_2_ or MX_2*y*_X_2(1–*y*)_^′^.^13,14^ For monolayers of Mo*_y_*W_1–*y*_S_2_ alloys, it was found that the Mo and W atoms are spatially distributed in a random way.^15^ Increasing the W content in samples of one or a few Mo*_y_*W_1–*y*_S_2_ layers caused a blue shift of the exciton peak in optical absorption and reflection spectra,^16,17^ in agreement with time-dependent density functional theory (TDDFT) calculations.^18^ According to DFT calculations, the valence band of monolayer Mo_0.5_W_0.5_S_2_ consists of atomic d-orbitals on both Mo and W atoms, while the conduction band consists predominantly of d-orbitals on Mo atoms.^19^

For optoelectronic applications, an understanding of electron–phonon and exciton–phonon interactions is important. The strength of these interactions governs charge transport,^20^ band gap renormalization,^21^ optical heating of the lattice,^22^ and intervalley scattering of excitons.^23−25^ In this regard, effects of temperature on optical absorption and photoluminescence spectra can provide information about the coupling strength between excitons and phonons in TMDCs.^23−26^

More than two decades ago, Ho et al.^27^ studied the effects of temperature on excitons in single crystals of Mo*_y_*W_1–*y*_S_2_ alloys by piezoreflectance measurements, which preferentially probe excitons near the sample surface. We extend these studies on the atomic layer deposited (ALD)^28^ bulk part of the Mo_0.6_W_0.4_S_2_ alloy to investigate the effects of temperature on peak energies and line widths of excitons. The almost equal content of Mo and W atoms in the alloy is of interest since it offers the possibility to realize intimate mixing of the transition metal atoms rather than having separate domains consisting of one atom type only. To elucidate the effects of the relative arrangement of Mo and W atoms in the alloy, we compared the measured spectra with results from ab initio TDDFT calculations. For this purpose, we constructed supercells having different positions of the metal atoms in the crystal structure of the alloy. The TDDFT calculations reproduced the experimental spectrum of the alloy for structures in which all layers contain both Mo and W atoms. In contrast, calculations on a structure containing W atoms in individual layers that are separated by layers containing only Mo atoms do not reproduce the experimental spectrum. From the latter, we infer that the ALD growth yields structures with a predominantly homogeneous spatial distribution of Mo and W atoms.

## 2 Methods

### 2.1 Temperature-Dependent Optical Transmission Measurements

We used our previously reported ALD procedure to grow thin films of MoS_2_, WS_2_, and the Mo_0.6_W_0.4_S_2_ alloy, with thicknesses of 6.3, 4.1, and 5.2 nm, respectively, on quartz substrates.^28^ The uncertainty in the fraction of Mo and W is ±0.01.^28^ The alloy was grown using an ALD supercycle length of two cycles (consisting of one MoS_2_ cycle and one WS_2_ cycle), to realize fine mixing of the Mo and W atoms. The composition was determined by X-ray photoelectron spectroscopy (XPS).^28^ The separation between adjacent layers in these materials is ∼0.6 nm, so the film thicknesses correspond to 10–11, 6–7, and 8–9 layers, respectively.

The optical transmission of the samples was measured using a home-built setup containing a DH-2000 halogen light source and an Ocean-optics Maya 2000 spectrometer. To vary the temperature, the samples were placed under vacuum in a He-closed cycle cryostat. These measurements yield the fraction of light transmitted, *T*, through the sample as a function of photon energy and temperature.

For the comparison of the optical properties of the samples with the optical absorption coefficient from TDDFT calculations (see Section 3.3), we determined the optical density (OD), using a PerkinElmer Lambda 1050 spectrometer with an integrating sphere. This could be done only at room temperature since the spectrometer was not equipped with a cryostat. Placing the sample in front of the light entrance of the integrating sphere yielded *T*, and placing it in the center provided *T* + *R*, where *R* is the fraction of light reflected. The results of 1 – *T*, *R*, and the fraction of light absorbed *A* = 1 – *R* – *T* are shown in Figure S1 for the pure compounds and the alloy. The optical density was obtained using the relation . The optical absorption coefficient, α, of a film with thickness *L* is related to the OD according to e^–α*L*^ = 10^–OD^, giving α = OD ln(10)/*L*.

### 2.2 TDDFT Calculations of Optical Absorption Coefficients

Electronic structure calculations were performed using the all-electron full-potential linearized augmented plane wave (LAPW) code Elk^29^ with PBE (GGA) functionals.^30^ For all materials, a hexagonal crystal structure (2H) was used, with experimental lattice constants of 3.169 and 12.324 Å for MoS_2_^31^ and 3.153 and 12.323 Å for WS_2_.^32^ A 2 × 2 × 1 supercell was constructed to study the Mo_0.625_W_0.375_S_2_ alloy with lattice parameters of 6.338 and 12.324 Å obtained by doubling the MoS_2_ unit cell. This is the smallest supercell describing the experimentally studied alloy with a composition very close to the experimental uncertainty (see Section 2.1). Note that larger supercells can be constructed. However, we only considered the 2 × 2 × 1 supercell because larger supercells require significantly more computational time (at least 1 order of magnitude) and more computer memory. The calculation of the dielectric response functions from TDDFT required a dense *k*-point grid to sample the Brillouin zone (BZ), hence a *k*-point grid of 16 × 16 × 8 for the primitive unit cell and an 8 × 8 × 8 *k*-point grid for the supercell were used. The set of LAPW basis functions was defined by specifying a cutoff parameter |***k*** + ***G***|_max_ whose value was set to 7.0 Bohr^–1^. Additionally, the response was calculated using ***G*** vectors of 1.5 Bohr^–1^ length. The number of conduction bands included in the calculations was 24 for both MoS_2_ and WS_2_ and 96 for the alloy.

In TDDFT, a Dyson-like equation was solved to obtain the dielectric response function^33^ whose real and imaginary parts can be used to obtain the optical absorption coefficient α.^34^ The method to obtain optical response functions was a two-step procedure. First, a ground-state calculation was done to obtain the converged density and potentials. Next, the dielectric functions of MoS_2_, WS_2_, and the Mo_0.625_W_0.375_S_2_ alloy were calculated as a function of photon energy using the bootstrap kernel,^35^ as it was capable of capturing excitons in the TDDFT calculations. The dielectric functions thus obtained were broadened by 80 meV for MoS_2_ and WS_2_ and 54 meV for the alloy to obtain the best matches with the experimental optical absorption coefficient spectra (α). Note that the broadening thus introduced in the calculated spectra did not explain the exciton line widths in the experimental spectra.

The absolute values of exciton energies with respect to the ground state cannot be accurately captured by TDDFT, due to the well-known band gap problem. To overcome this, we employed the so-called “scissor operator” method that shifts the entire optical absorption spectrum (α) in energy. To reproduce the lowest experimental exciton energy, we used energy shifts of 0.03, 0.08, and 0.06 eV for MoS_2_, WS_2_, and the Mo_0.625_W_0.375_S_2_ alloy, respectively.

## 3 Results and Discussion

### 3.1 Optical Transmission Spectra

**Figure 1:**
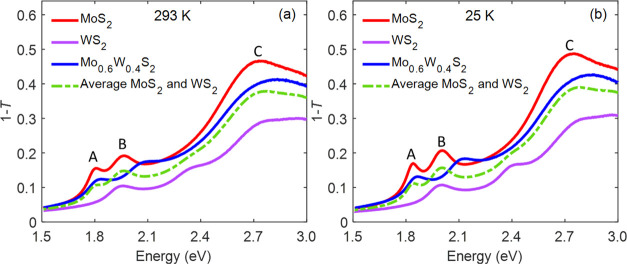
(a) Room-temperature (293 K) and (b) low-temperature (25 K) optical transmission spectra of MoS_2_ (red), WS_2_ (magenta), and the Mo_0.6_W_0.4_S_2_ alloy (blue). The dashed green curves are the average of the MoS_2_ and WS_2_ spectra.

Figure 1a shows the optical transmission spectra of MoS_2_, WS_2_, and the Mo_0.6_W_0.4_S_2_ alloy at room temperature (293 K). These spectra show the magnitude of 1 – *T*, which is the fraction of incident light that is not transmitted through the sample. The spectra of MoS_2_ and WS_2_ agree with previous results.^8,36^ Two distinct peaks (marked by A and B) can be seen in all three materials. The peaks are due to photoexcitation from the ground state to A and B exciton states. The energies of these peaks are determined by spin–orbit coupling and interlayer interactions at the *K* and *K*′ points of the Brillouin zone (BZ).^37−39^ Toward the higher energy side, a broad absorption feature is observed (often addressed as C exciton), which originates from multiple transitions from the highest valence band to the lowest conduction bands near the Γ point of the BZ.^40^ On lowering the temperature to 25 K (Figure 1b), the exciton peaks of all three materials become narrower and shift to higher energy.

To gain qualitative insights into the effect of alloying, we also show the average of the spectra of pure MoS_2_ and WS_2_ as green dashed curves in Figure 1 (a quantitative comparison of the measured OD and the results from TDDFT is discussed in Section 3.3). The average spectra at 293 and 25 K both differ from the spectra of the alloy. Most strikingly, the B exciton peak of the alloy appears at significantly higher energy than in the average spectra. These differences indicate that formation of excitons in domains consisting of either predominantly MoS_2_ or WS_2_ is unlikely. As a consequence, the probability that photoexcitation leads to formation of a charge transfer exciton at a boundary between these material domains is small. Indeed, the peak of the A exciton in the alloy spectrum appears at higher energy than that in the spectrum of MoS_2_, while that of a charge transfer exciton would be at lower energy.

Inspection of the transmission spectra of the alloy points toward closer similarities to MoS_2_ than to WS_2_. Despite intimate mixing and nearly equal Mo and W content in the alloy,^28^ the energies of the A and B excitons in the alloy are closer to those of pure MoS_2_, as also found for samples of one or a few Mo_0.5_W_0.5_S_2_ layers before.^16,17^ This suggests that the wave functions of excitons in the Mo_0.6_W_0.4_S_2_ alloy have a larger amplitude on Mo atoms than that on W atoms. The latter agrees with charge density distributions for the highest valence and lowest conduction band states obtained from DFT calculations.^14,19^ Interestingly, according to our TDDFT calculations, the mutual arrangement of Mo and W atoms within the material has a large impact on the shape of the optical absorption spectrum (see Section 3.3). To gain insights into the nature of exciton–phonon coupling, we first proceed with a discussion of the measured effects of temperature on exciton peak positions and line widths in Section 3.2.

### 3.2 Temperature Dependence of A and B Exciton Peak Energies and Line Widths

To gain further insights into the relative contributions of Mo and W atoms to the character of excitons, we compare the effects of exciton–phonon coupling in the Mo_0.6_W_0.4_S_2_ alloy with those in MoS_2_ and WS_2_. We studied exciton–phonon coupling by the analysis of the temperature dependence of exciton peak energies and line widths in the transmission spectra, as outlined in Section 2 in the Supporting Information. The peaks due to A and B excitons could each be described by a Lorentzian function with line width Γ_X_, (where X = A, B), which is defined as the full width at half-maximum (FWHM), see eq S1. The contribution of optical reflection, below band gap absorption due to defects,^41^ and the broad C absorption feature at higher energy in the optical transmission spectra in Figure 1 could be described by two Gaussian functions. The total fit function thus consists of two Lorentzian and two Gaussian functions, see eq S1. Figure S2 shows that the fits reproduce the experimental transmission spectra very well.

**Figure 2:**
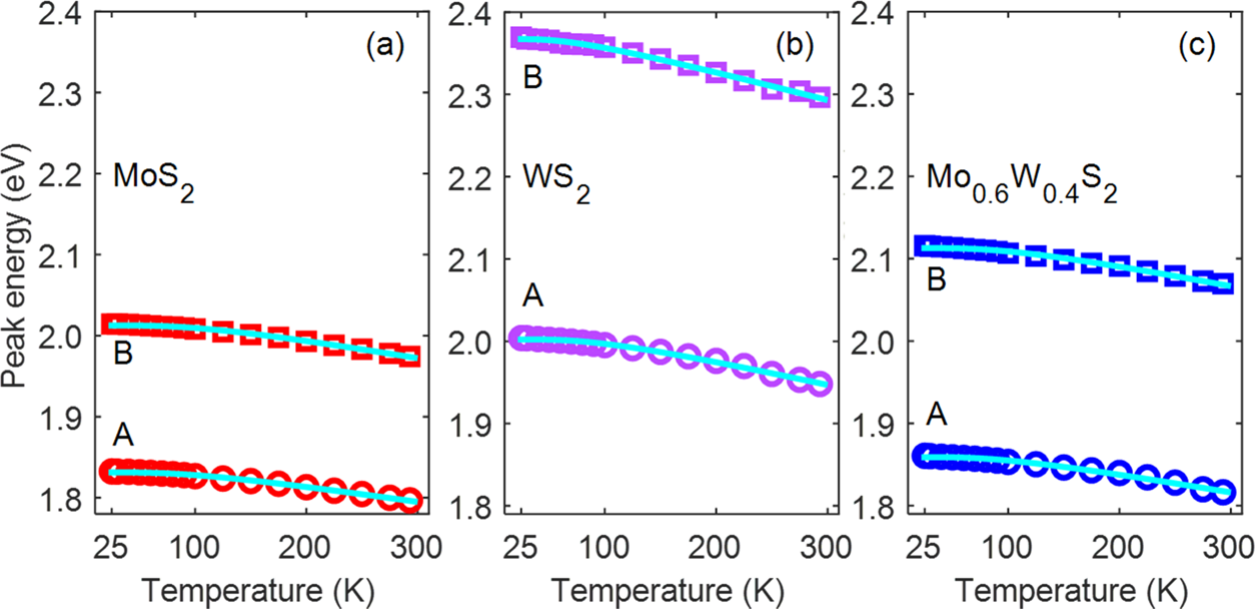
Temperature dependence of the A and B exciton peak energies for (a) MoS_2_, (b) WS_2_, and (c) the Mo_0.6_W_0.4_S_2_ alloy, obtained from the measured transmission spectra (markers). The solid cyan curves are fits of eq 1 to the experimental peak energies.

**Figure 3:**
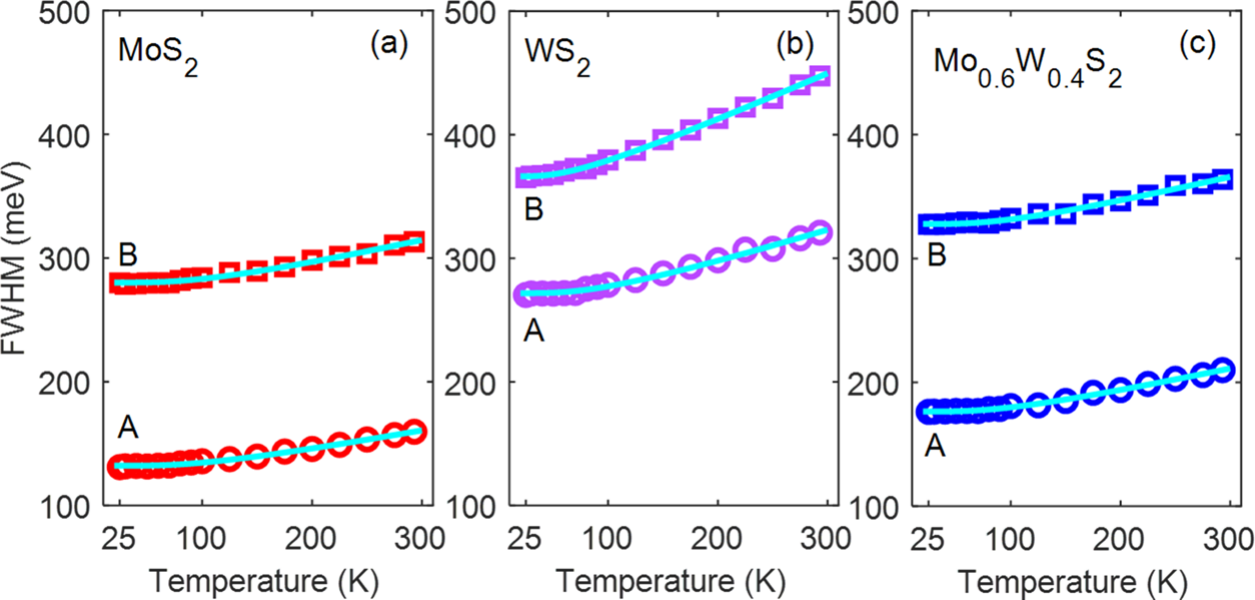
Temperature dependence of the line widths (FWHM, markers) of the A and B exciton peaks for (a) MoS_2_, (b) WS_2_, and (c) the Mo_0.6_W_0.4_S_2_ alloy. The solid cyan lines are fits to the experimental data.

Figures 2 and 3 show the temperature dependence of the A and B exciton peak energies and line widths, as obtained from fits of eq S1 to the experimental transmission spectra. At all temperatures, the peak energies and line widths of the Mo_0.6_W_0.4_S_2_ alloy are closer to those of MoS_2_ than those of WS_2_. This further supports the idea that excitons have more Mo than W character, as we already inferred above from Figure 1.

The decrease of the exciton peak energies with increasing temperature is due to the availability of more phonons at higher temperatures that can be absorbed upon photoexcitation from the electronic ground state to an exciton state, as well as electron–phonon coupling due to interaction between the motion of electrons and atomic nuclei (change of bond lengths and breakdown of the Born–Oppenheimer approximation).^42−44^ Following previous studies,^26,44−46^ we describe the temperature dependence of the exciton peak energies by the following semiempirical O’Donnell equation^47^
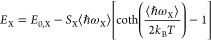
1where X = A, B denotes the exciton type, and *k*_B_ and ℏ are the Boltzmann and the reduced Planck constant, respectively. In eq 1, *E*_0,X_ is the exciton peak energy at zero temperature, *S*_X_ is a dimensionless constant that increases with the exciton–phonon coupling strength, and ⟨ℏω_X_⟩ is the coupling-weighted average of the phonon energies that interact with the exciton.^48^

Fits of eq 1 to the A and B exciton peak energies with *E*_0,X_, *S*_X_, and ⟨ℏω_X_⟩ as adjustable parameters are shown as solid cyan curves in Figure 2. Equation 1 reproduces the temperature dependence of the exciton peak energies very well and the values of the fit parameters are presented in Table 1. The exciton peak energies *E*_0,A_ and *E*_0,B_ for the alloy are closer to those for MoS_2_ than for WS_2_. In addition, the fitted values of *S*_A_ and *S*_B_ (near 1.5) for the alloy are similar to those of MoS_2_, while they are about 25% smaller than the values obtained for WS_2_ (near 2.0). These findings corroborate our notice in Section 3.1 that exciton wave functions in the alloy have a larger amplitude on Mo atoms than that on W atoms so that the former has a predominant effect on exciton–phonon coupling. Within the experimental uncertainty, the average phonon energies ⟨ℏω_X_⟩ for both A and B excitons are similar for all three materials and are close to the value of 22.1 meV reported for MoS_2_ and WS_2_ in the literature.^49,50^

**Table 1:** Fitted Values of the Exciton–Phonon Coupling Strength, *S*_X_, the Average Phonon Energy, ⟨ℏω_X_⟩, Inhomogeneous Line Width Broadening, Γ_X,I_, and the Exciton–Phonon Interaction Strength, Γ_X,ph_, for MoS_2_, WS_2_, and the Mo_0.6_W_0.4_S_2_ Alloy

	MoS_2_	WS_2_	Mo_0.6_W_0.4_S_2_
*E*_0A_ (eV)	1.80 ± 0.01	1.96 ± 0.01	1.83 ± 0.01
*E*_0B_ (eV)	1.97 ± 0.01	2.34 ± 0.01	2.09 ± 0.01
*S*_A_	1.4 ± 0.2	1.9 ± 0.2	1.5 ± 0.1
*S*_B_	1.5 ± 0.1	2.1 ± 0.1	1.6 ± 0.1
ℏ*ω*_A_ (meV)	26.4 ± 2.2	22.8 ± 3.1	24.4 ± 2.5
ℏ*ω*_B_ (meV)	26.4 ± 1.9	16.4 ± 3.5	24.4 ± 1.5
Γ_A,I_ (meV)	132.1 ± 0.4	271.8 ± 0.9	176.4 ± 0.1
Γ_B,I_ (meV)	280.2 ± 0.7	366.2 ± 0.6	327.8 ± 0.1
Γ_A,ph_ (meV)	50.8 ± 1.8	72.4 ± 3.0	54.6 ± 1.9
Γ_B,ph_ (meV)	60.8 ± 3.1	73.9 ± 1.6	59.7 ± 2.5

We analyze the temperature dependence of the line widths of the Lorentzians in eq S1 of the A and B exciton peaks by using the following expression^51^
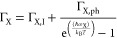
2The first term at the right-hand side of eq 2, Γ_X,I_, represents inhomogeneous line width broadening induced by temperature-independent mechanisms, such as scattering of excitons on structural defects or impurities. The second term describes exciton–phonon scattering for both absorption and emission of phonons. The average energies of phonons that couple with excitons, ⟨ℏω_X_⟩, were taken equal to the values obtained from fitting eq 1 to the peak energies, see Table 1.

The solid cyan lines in Figure 3a–c are the least-squares fits of eq 2 to the FWHM values, with the latter obtained from fits of eq S1 to the optical transmission spectra in Figure S2. The results for the inhomogeneous broadening, Γ_X,I_, and the broadening due to exciton–phonon scattering, Γ_X,ph_, are presented in Table 1. For each of the three materials, the values of the inhomogeneous broadening of the A exciton, Γ_A,I_, are smaller than those of the B exciton, Γ_B,I_, similar to results for single crystals.^52^ Interestingly, the values of both Γ_A,ph_ and Γ_B,ph_ of the alloy are close to the corresponding values for MoS_2_, while they are significantly lower than those for WS_2_. This is in line with the exciton peak energies and the values of *S*_A_ and *S*_B_ for the alloy being nearest to those of MoS_2_, as discussed above. The larger exciton–phonon scattering rate for B excitons can be due to the additional ultrafast decay channel of B excitons involving their relaxation to A excitons by emission of phonons, as discussed previously.^53^

Our values of *E*_0,X_, *S*_X_, ⟨ℏω_X_⟩, and Γ_X,ph_ for ALD-grown MoS_2_ and WS_2_ films are within the range reported for mono- or few-layer TMDC samples that were obtained by mechanical exfoliation or chemical vapor deposition (CVD)^26,45,46,50,54−56^ and CVD-grown bulk samples.^27,50^ Note that the values of these parameters can vary from one sample to another due to differences in sample preparation, dielectric environment (in particular for mono- and few-layer samples), etc. Our values for the inhomogeneous line width broadening, Γ_X,I_, are higher than those that Ho et al.^27^ obtained from temperature-dependent piezoreflectance measurements on CVD-grown crystals of MoS_2_, WS_2_, and Mo*_x_*W_1–*x*_S_2_ alloys. This may result from a larger degree of structural disorder in our ALD-grown samples. Indeed the grain size in ALD-grown samples is ∼10 nm, which is much smaller than that for CVD-grown crystals.^57^ Interestingly, the values of the exciton–LO phonon coupling strength, Γ_X,ph_, reported by Ho et al.^27^ are a factor of 2–3 higher than ours. This could be due to the fact that their piezoreflectance measurements probe excitons near the sample surface, which would then appear to couple to surface phonons with higher strength than the bulk exciton–phonon coupling probed in our experiments.

### 3.3 TDDFT Calculations of the Optical Absorption Spectrum

**Figure 4:**
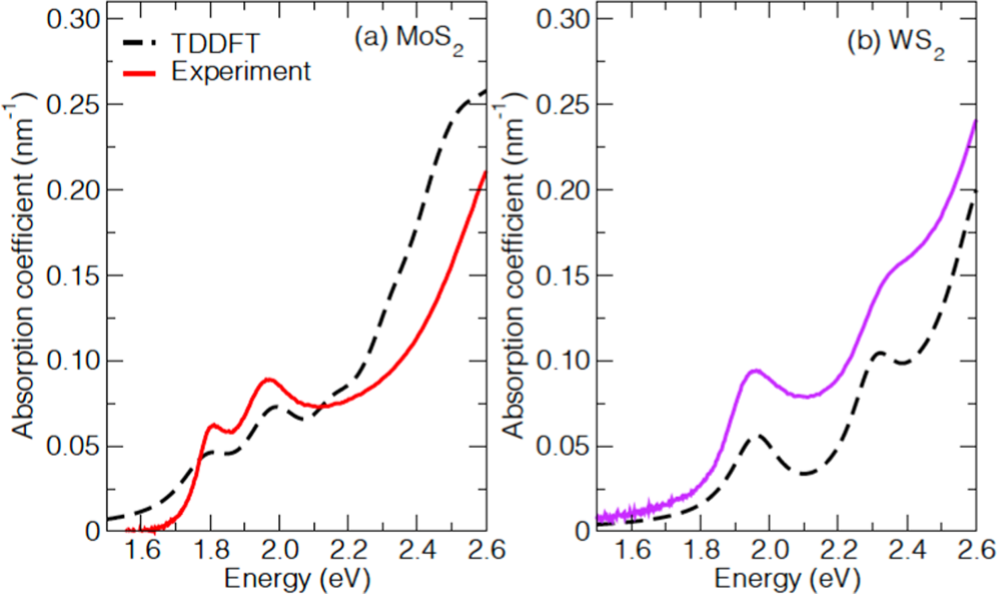
Absorption coefficient, α, obtained from TDDFT calculations (black dashed curves) together with the experimental results at 293 K for (a) MoS_2_ and (b) WS_2_.

The real and imaginary parts of the dielectric functions obtained from the TDDFT calculations are shown in Figures S3–S5 and these were used to calculate the optical absorption coefficient, α, according to eq S3. The calculated absorption coefficients for MoS_2_ and WS_2_ are shown in Figure 4, together with the experimental data at 293 K. The optical absorption coefficients were obtained, as described in Section 2.2, using the spectra of *T* and *R* in Figure S1. The calculations reproduce the relative energies of the A and B excitons very well, see also Table 2. In addition, the calculations reproduce the magnitude of the optical absorption coefficient to within a factor 2.

**Table 2:** Energies of the A and B Excitons in MoS_2_, WS_2_, and the Mo_0.6_W_0.4_S_2_ Alloy^a^

	MoS_2_	WS_2_	Mo_0.6_W_0.4_S_2_	Mo_0.625_W_0.375_S_2_
*E*_A_ (exp.) (eV)	1.80	1.96	1.83	
*E*_B_ (exp.) (eV)	1.97	2.34	2.09	
*E*_B_ – *E*_A_ (exp.) (meV)	170	380	260	
*E*_B_ – *E*_A_ (TDDFT calc.) (meV)	179	353		255

a The last two rows show the energy difference between the exciton energies from experiments (exp.) and the TDDFT calculations.

**Figure 5:**
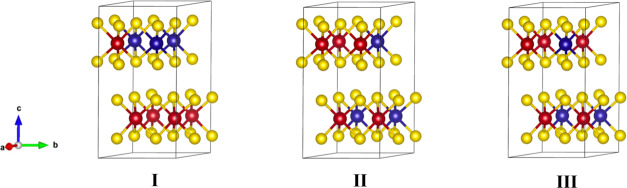
Three physically distinct arrangements of atoms in the Mo_0.625_W_0.375_S_2_ alloy. Each 2 × 2 × 1 supercell of the Mo_0.625_W_0.375_S_2_ alloy shows the different arrangements of metal and chalcogen atoms where the Mo atoms are red, the W atoms are blue, and the S atoms are yellow. Heterogeneous structure I has alternating layers of Mo atoms only and layers containing both Mo and W atoms. In homogenous structures II and III, all layers contain Mo and W atoms.

As discussed in Section 2, we describe the Mo_0.6_W_0.4_S_2_ alloy by a periodic crystal structure with the smallest possible (2 × 2 × 1) supercell, resulting in the Mo_0.625_W_0.375_S_2_ alloy, see Figure 5. One unit cell then contains 5 Mo atoms, 3 W atoms, and 16 S atoms that are arranged in two layers bonded by van der Waals forces. By permutation of the 5 Mo and 3 W atoms, one can realize 28 different arrangements. These can be categorized into two groups: (1) 4 “heterogeneous” structures in which every other layer contains only Mo atoms and the layers in between contain also W atoms, and (2) 24 “homogeneous” structures in which both layers contain Mo and W atoms. Applying the symmetry operations of translation, rotation, mirror planes, and their combinations, we obtain three physically distinct structures (I, II, and III), as shown in Figure 5.

**Figure 6:**
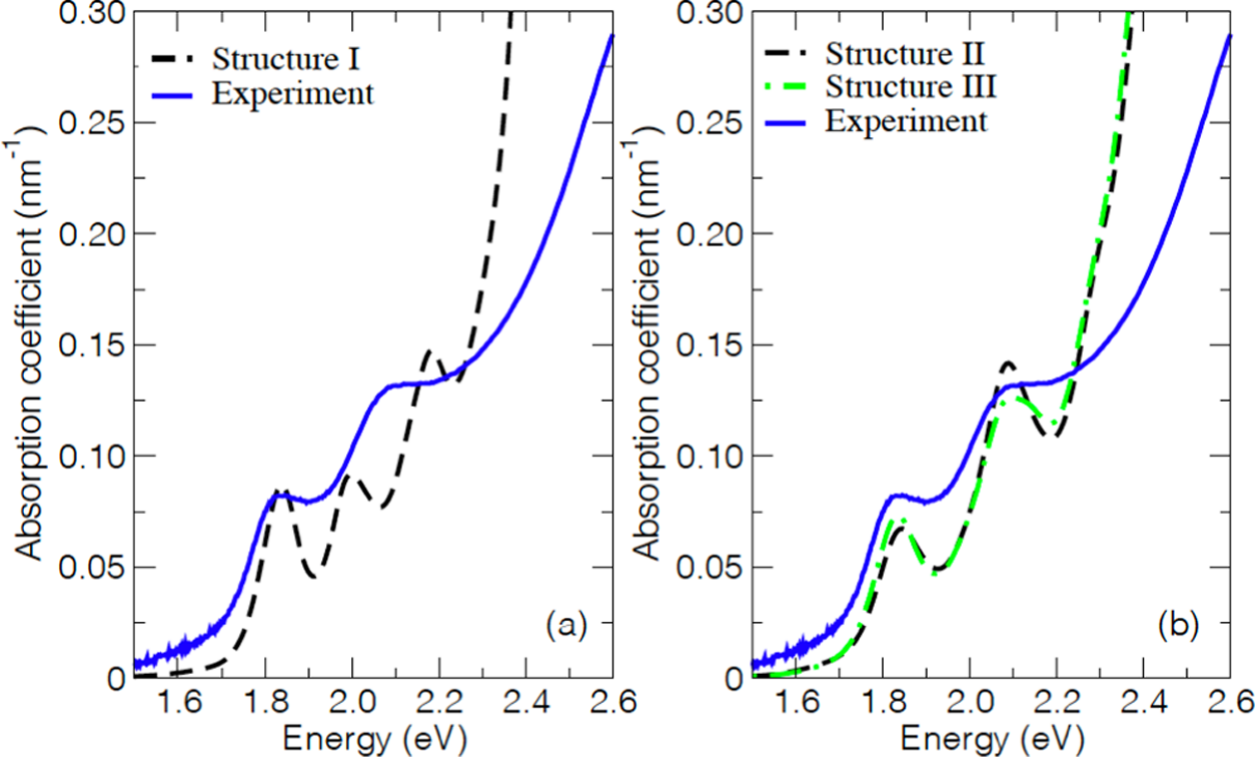
Calculated absorption coefficient of (a) structure I and (b) structures II and III of the Mo_0.625_W_0.375_S_2_ alloy, together with the experimental spectrum at room temperature (293 K).

The calculated optical absorption coefficient of the Mo_0.625_W_0.375_S_2_ alloy with heterogeneous structure I is shown in Figure 6a, together with the experimental spectrum. The presence of three peaks in the calculated spectrum disagrees with the two excitonic peaks in the experimental spectrum. We suspect, but cannot prove here, that the peak at the lowest energy calculated for structure I is due to excitons having some more charge transfer character than the peaks at higher energy. For such excitons, the electron would have a somewhat larger probability to reside on Mo atoms, while the hole is preferentially present on W atoms. Interestingly, the calculated spectra of structures II and III shown in Figure 6b agree with the experimental spectrum. The relative energies of the A and B excitons, as well as the magnitude of the optical absorption coefficient, are very well reproduced by these structures (see Table 2). From this, we infer that the Mo and W atoms in the ALD-grown films are to a large extent mixed homogenously, as in structures II and III. This agrees with the previously reported random arrangement of Mo and W atoms in monolayers of these alloys grown by chemical vapor transport.^14,15^ The very different result from TDDFT calculations for structure I in Figure 6a compared with those for structures II and III in Figure 6b shows that the mutual arrangement of Mo and W atoms has a strong effect on the optical absorption spectrum.

Unfortunately, the TDDFT calculations performed with the Elk code do not provide the atom resolved composition of the exciton wave functions, and therefore, we cannot obtain the distribution of the electron and hole within an exciton among the atoms. To investigate the spatial distribution of the electron and the hole within an exciton, calculations at a higher level of theory are needed, e.g., by describing excitons on the basis of the Bethe–Salpeter equation.^58^

## 4 Conclusions

We performed a combined experimental and time-dependent density functional theory (TDDFT) study of the optical absorption/transmission spectra of ALD-grown thin films of MoS_2_, WS_2_, and the Mo_0.6_W_0.4_S_2_ alloy. The temperature dependence of the peak energies and line widths of the A and B excitons in the alloy is close to that for MoS_2_. This suggests that the exciton wave functions have a larger amplitude on Mo atoms than that on W atoms. From the comparison of the measured optical absorption spectra with those from TDDFT calculations, we infer that Mo and W atoms are homogeneously distributed throughout the alloy. Further, the mutual arrangement of Mo and W atoms in the material has a strong effect on the shape of the optical absorption spectrum. These results provide clear support toward structural engineering of two-dimensional van der Waals materials through atomic arrangements, extending the already rich variety of properties in this class of materials.

## Supporting Information Available

Absorption spectra; fits to temperature-dependent optical transmission spectra of MoS_2_, WS_2_, and the Mo_0.6_W_0.4_S_2_ alloy; and relation between absorption coefficient and dielectric function (PDF)
