# Supplementary material for: Effects of the Structure and Temperature on the Nature of Excitons in the Mo0.6W0.4S2 Alloy
Source: J Phys Chem C Nanomater Interfaces. 2022 Jan 25;126(4):1931–8. doi: 10.1021/acs.jpcc.1c09806 (PMC8819651; doi:10.1021/acs.jpcc.1c09806)
Supplement: Supplementary file 1 — jp1c09806_si_001.pdf [file jp1c09806_si_001.pdf]

Effects of Structure and Temperature on the Nature of Excitons in the $Mo_{0.6}W_{0.4}S_2$ Alloy

AUTHORS

Deepika Poonia,[†] Nisha Singh,[†] Jeff J.P.M. Schulpen,[‡] Marco van der Laan,[¶] Sourav Maiti,[†] Michele Failla,[†] Sachin Kinge,[§] Ageeth A. Bol,[‡] Peter Schall,[¶] and Laurens D.A. Siebbeles*[†]

AFFILIATIONS

[†]*Optoelectronic Materials Section, Department of Chemical Engineering, Delft University of Technology, 2629 HZ Delft, The Netherlands*

[‡] *Department of Applied Physics, Eindhoven University of Technology, P.O. Box 513, 5600 MB Eindhoven, The Netherlands*

[¶] *Institute of Physics, University of Amsterdam, 1098 XH Amsterdam, The Netherlands*

[§]*Materials Research & Development, Toyota Motor Europe, B1930 Zaventem, Belgium*

1. Transmission spectra of MoS_2, WS_2, and the $Mo_{0.6}W_{0.4}S_2$ alloy together with the fractions reflected and absorbed light.

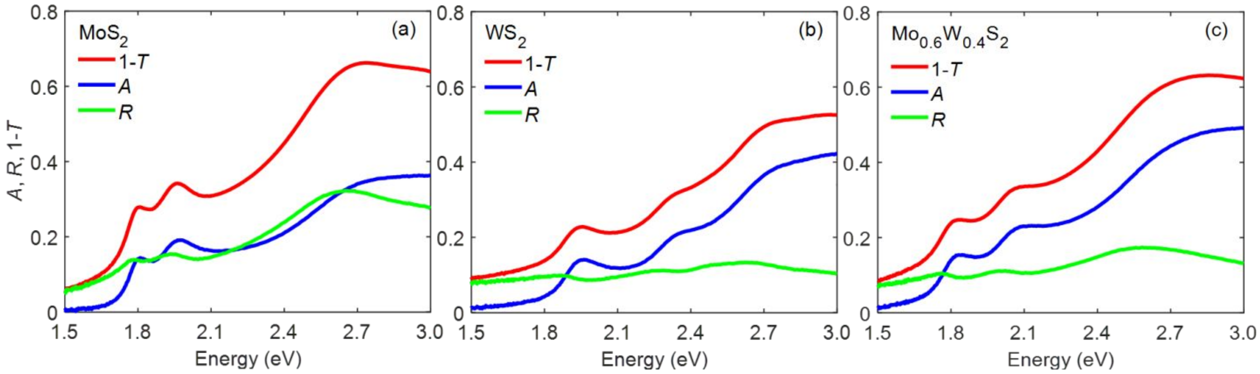

Figure S1. Room temperature transmission spectra (1-*T*) and fractions reflected (*R*) and absorbed ($A = 1 - R - T$) light for MoS_2, WS_2 and the $Mo_{0.6}W_{0.4}S_2$ alloy.

2. Fits to the temperature-dependent transmission spectra. The optical transmission spectra were analyzed by fitting Equation S1 to the experimental results

$$1 - T(E) = \sum_{i=A,B} \frac{1}{2\pi} \frac{C_i \Gamma_i}{(E - E_i)^2 + (\Gamma_i/2)^2} + \sum_{j=1}^{2} \frac{C_j}{\sigma_j \sqrt{2\pi}} e^{\frac{-(E-E_j)^2}{2\sigma_j^2}} \qquad \text{S1}$$

with *E* the photon energy *E.* The two Lorentzian functions with amplitude C_i and linewidth Γ_i describe the A and B exciton peaks. The Gaussian functions describe optical reflection, below band gap absorption due to defects and the broad C absorption feature at higher energy. Figure S2 shows the fit results together with the experimental spectra.

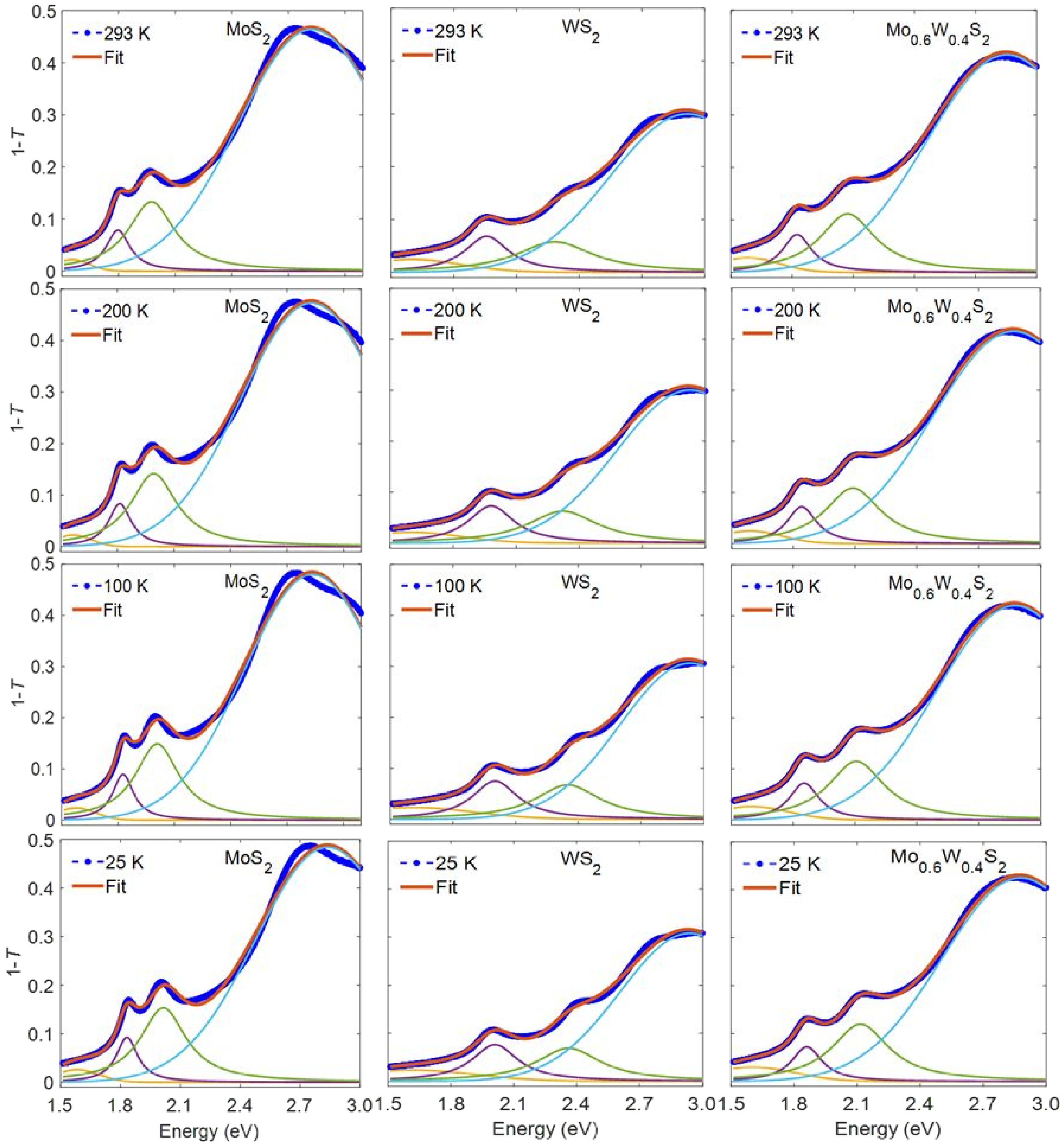

Figure S2. Fits to the experimental transmission spectra of MoS_2, WS_2, and the $Mo_{0.6}W_{0.4}S_2$ alloy at 293 K, 200 K, 100 K, and 25 K, respectively. The dark blue curves are the experimental spectra, the red curve is the fit of Equation S1 with the Lorentzian functions shown in purple and green and the Gaussians in yellow and light blue.

3. Relation between absorption coefficient and dielectric function. The optical absorption coefficient, α, can be obtained from the real (ε_1) and imaginary (ε_2) parts of the dielectric function using the following relations[1].

$$n = \sqrt{\frac{1}{2}[[\varepsilon_1^2 + \varepsilon_2^2]^{1/2} + \varepsilon_1]}\,, \quad k = \sqrt{\frac{1}{2}[[\varepsilon_1^2 + \varepsilon_2^2]^{1/2} - \varepsilon_1]} \qquad \text{S2}$$

and

$$\alpha = 2\omega k/c\,, \qquad \text{S3}$$

where n is the refractive index, k the extinction coefficient and ω the radian frequency of the light, respectively.

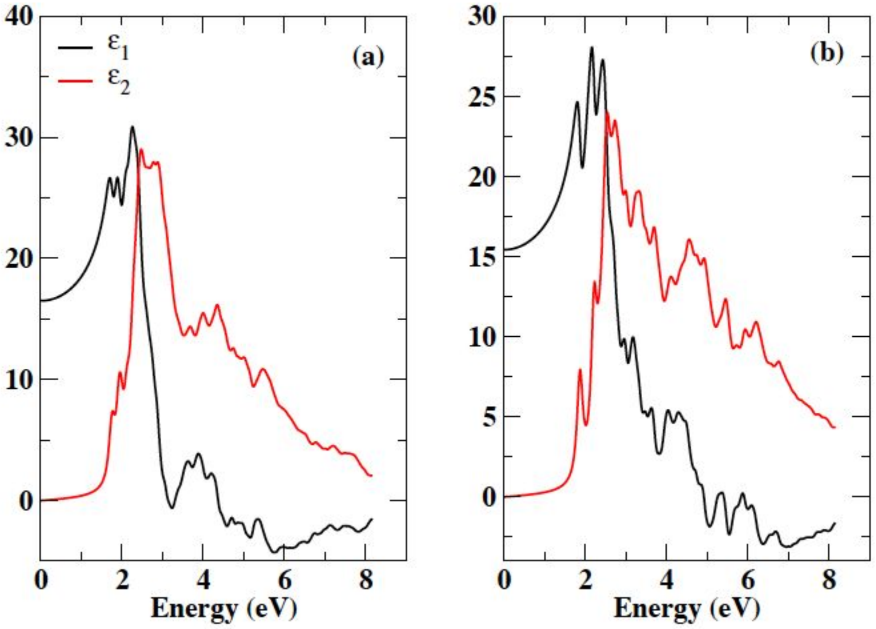

Figure S3. The real and imaginary part of the dielectric function obtained from the TDDFT calculations for (a) MoS_2 and (b) WS_2.

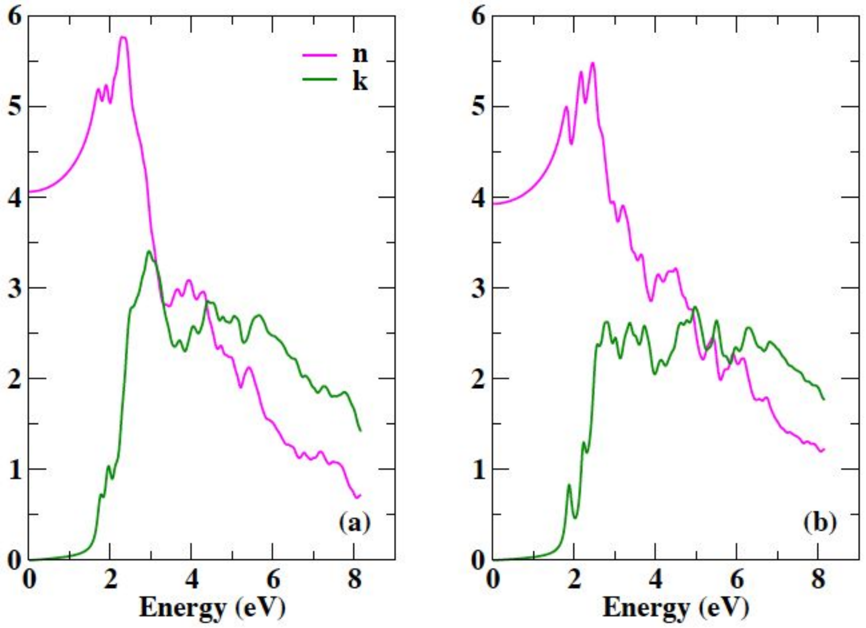

Figure S4. The refractive index and extinction coefficient obtained from Equation S2 for (a) MoS_2 and (b) WS_2.

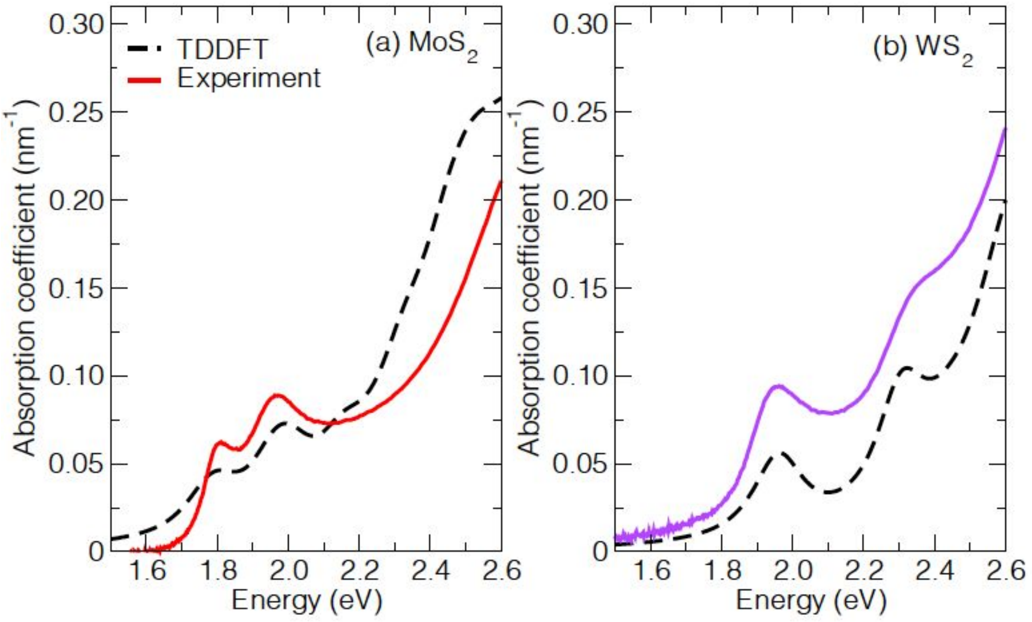

Figure S5. The absorption coefficient obtained from Equation S3 for (a) MoS_2 and (b) WS_2. The TDDFT results have not been shifted in energy.

REFERENCES

(1) Wooten, F. *Optical Properties of Solids, Academic Press,* New York and London, 1972.
